# A novel practical method to predict anterior cruciate ligament hamstring graft size using preoperative MRI

**DOI:** 10.1186/s43019-024-00216-7

**Published:** 2024-04-04

**Authors:** Zi Qiang Glen Liau, Matthew Song Peng Ng, Shawn Shao En Low, Brian Zhaojie Chin, James Hoi Po Hui, Fareed Husain Yusuf Kagda

**Affiliations:** 1 Department of Orthopaedic Surgery, National University Hospital, 5 Lower Kent Ridge Rd, Singapore, 119074 Singapore; 2 Yong Loo Lin School of Medicine, National University of Singapore, Singapore, Singapore; 3 Department of Orthopaedic Surgery, Ng Teng Fong General Hospital, Singapore, Singapore

**Keywords:** Knee, ACL, MRI, Graft choice

## Abstract

**Background:**

Predicting hamstring graft size preoperatively for anterior cruciate ligament (ACL) reconstruction is important for preempting an insufficient diameter in graft size intraoperatively, possibly leading to graft failure. While there are multiple published methods using magnetic resonance imaging (MRI) picture archiving and communication systems (PACS), most are not feasible and practical. Our study aims to (1) practically predict the ACL hamstring graft size in a numerically continuous manner using the preoperative MRI from any native MRI PACS system, (2) determine the degree of correlation between the predicted and actual graft size, and (3) determine the performance of our prediction method if we define an adequate actual graft size as ≥ 8 mm.

**Methods:**

A retrospective review of 112 patients who underwent primary ACL reconstruction with quadrupled hamstring semitendinosus-gracilis grafts at a tertiary institution was conducted between January 2018 and December 2018. Graft diameter can be predicted in a numerically continuous manner as √[2*(AB + CD)], where A and B are the semitendinosus cross-sectional length and breath, respectively, and C and D are the gracilis cross-sectional length and breath, respectively.

**Results:**

A moderately positive correlation exists between the predicted and actual graft diameter (*r* = 0.661 and *p* < .001). Our method yields a high specificity of 92.6% and a moderate sensitivity of 67.2% if we define an adequate actual graft size as ≥ 8 mm. An area under receiver-operating characteristic curve shows good discrimination (AUC = 0.856).

**Conclusions:**

We present a practical method to predict the ACL hamstring graft size with high specificity using preoperative MRI measurements.

**Supplementary Information:**

The online version contains supplementary material available at 10.1186/s43019-024-00216-7.

## Background

Graft options for ACL reconstruction include autograft iliotibial (IT) band, quadrupled hamstrings (QHS), bone–patellar tendon–bone (BTB), quadriceps tendon (QT), allografts, and hybrid grafts, with the QHS graft being one of the most widely used [1, 2]. Predicting hamstring graft size preoperatively is important, as it may help preempt an insufficient diameter in graft size intraoperatively, which may lead to graft failure. To overcome this, the orthopedic surgeon may either use an allograft from the tissue bank, adopt a different surgical technique, extract a new graft from the patient (bone–patellar tendon–bone or quadricep autograft or allograft), or consider using a triple graft or augment/tape [3]. This helps in providing better preoperative counseling and more accurate costing quotations to patients.

Multiple studies demonstrate that there is a higher risk of graft failure or rerupture if the graft is less than 8 mm in diameter [4–6], while a systematic review confirms this finding as well [7]. While there are multiple published models for the prediction of the hamstring graft preoperatively, most are not feasible and practical for the following reasons: recruiting the help of a radiologist for measurement [8–11], usage of specialized three-dimensional software or the freehand region of interest (lasso) tool that is not widely available on all MRI PACS systems [8–12], and using 3T MRIs to predict the measurement [11, 13], which is costly and not commonly performed.

Our study aims to (1) practically predict the ACL hamstring graft size in a numerically continuous manner, by surgeons or surgical assistants of all levels of training, using the preoperative MRI from any native MRI PACS system, (2) determine the degree of correlation between the predicted and actual graft size using our prediction method, and (3) determine the performance of our prediction method in terms of specificity, sensitivity, and discriminative ability, if we define an adequate actual graft size as ≥ 8 mm.

## Methods

**Fig. 1:**
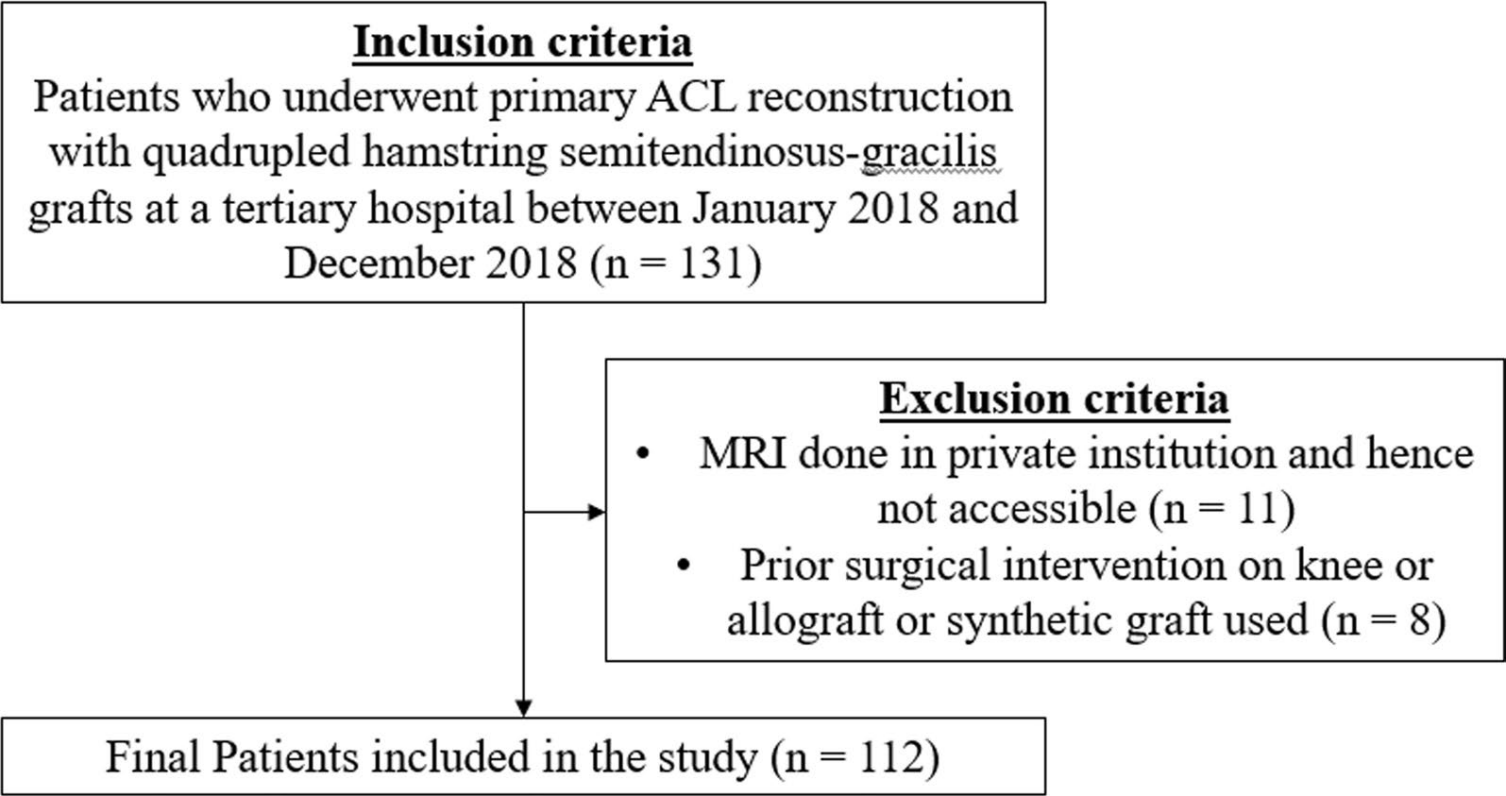
Flow diagram showing the inclusion and exclusion criteria for the study

A retrospective review of patients who underwent primary ACL reconstruction with quadrupled hamstring semitendinosus-gracilis (ST-G) grafts at a tertiary institution between January 2018 and December 2018 was conducted. A total of 131 patients were first identified from a surgical database comprising five surgeons with a minimum of 20 years of experience harvesting semitendinosus–gracilis grafts. Inclusion criteria were skeletally mature patients who underwent primary ACL reconstruction with quadrupled ST-G hamstring grafts, with intact hamstrings visible on preoperative MRI images. Exclusion criteria included patients who had their MRI done in private institutions rendering the images inaccessible, prior surgical intervention on the imaged knee, allograft ACL reconstruction, or synthetic graft ACL reconstruction (Fig. 1).

### Semitendinosus and gracilis tendon measurements

MRI imaging of the affected knees were performed in our tertiary institution by senior radiographers using a 1.5T Siemens MAGNETOM Aera scanner (Erlangen, Germany), with sequences including Sagittal PD/T2-weighted spin echo, coronal PD/T2-weighted spin echo, and axial medic. Two independent evaluators, a junior medical officer (second graduate year after medical school) and an orthopedic registrar (R5) with no prior radiology posting experience independently measured the cross-sectional lengths and breadths of both semitendinosus and gracilis grafts for all included patients. The evaluators were blinded to the actual graft sizes, which were measured intraoperatively using both sizing cylinders and graft sizing tubes in 0.5 mm increments. An instructional document outlining the measurement protocol and technique was produced for all the reviewers to use so that the measurement process would be standardized. Both evaluators received a similar 1 h training session, as detailed in the subsequent paragraph and were blinded to each other’s results.

**Fig. 2:**
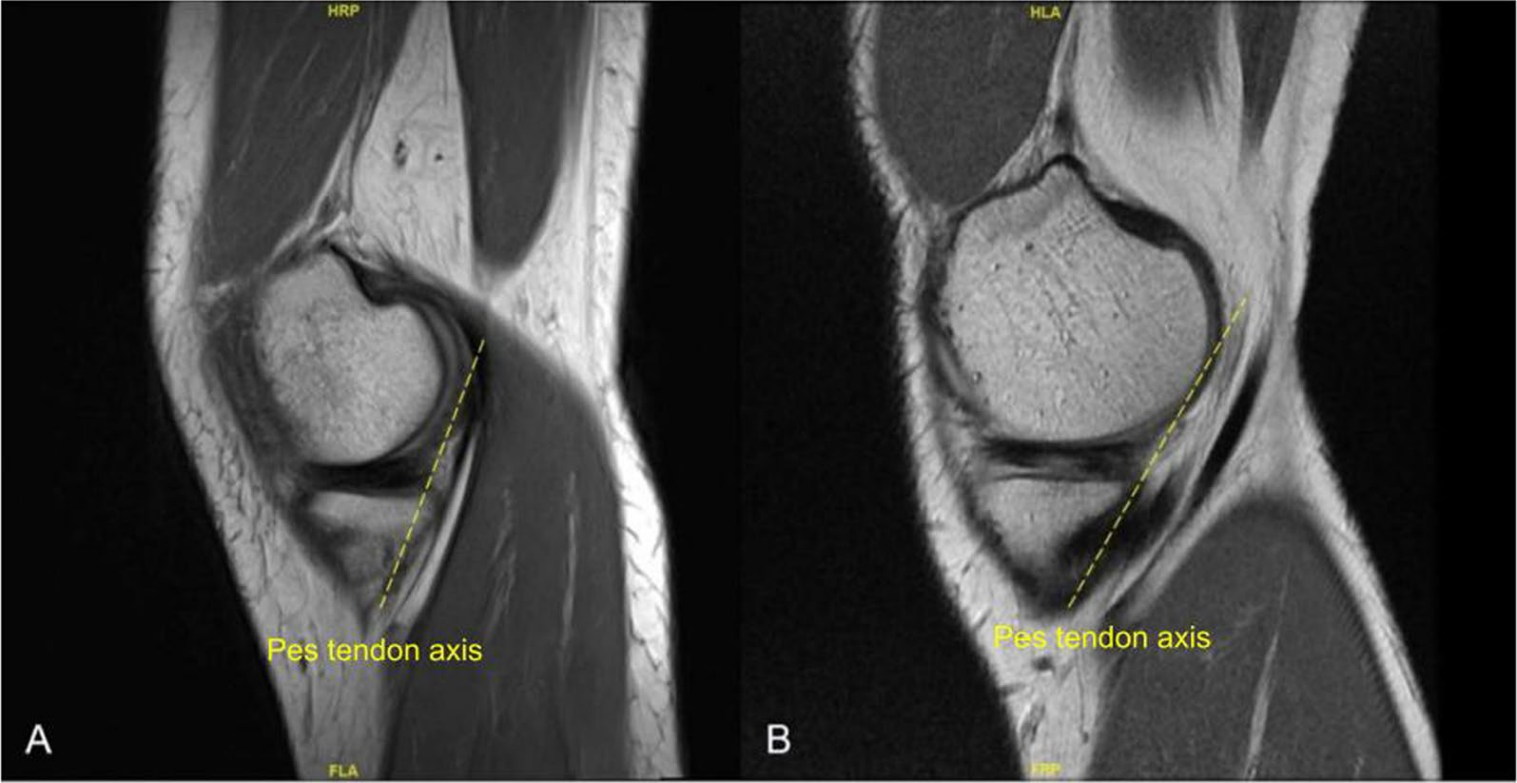
T2-weighted MRI showing sagittal cuts of the knee of two patients, demonstrating differing axes of pes tendons which are used as reference for subsequent measures

**Fig. 3:**
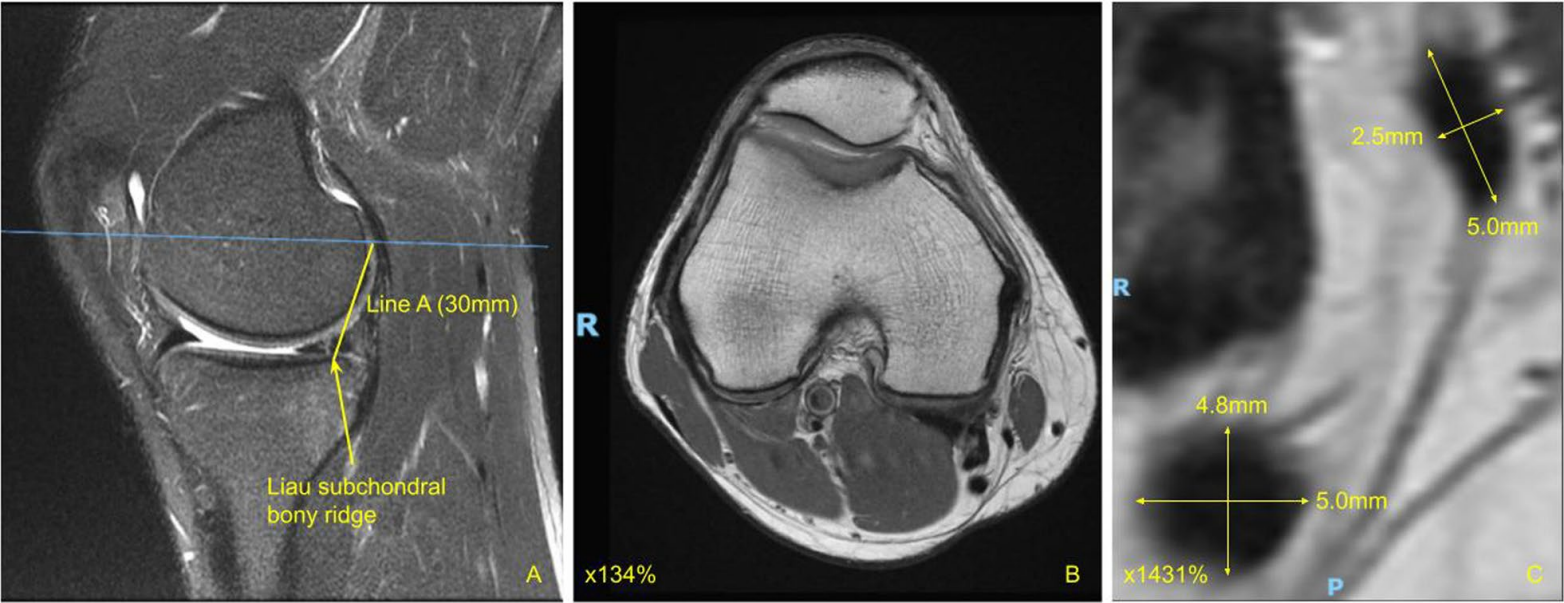
T2-weighted MRI showing sagittal and axial cuts of the affected knee

The measurement protocol was as follows: measurements of both semitendinosus and gracilis grafts on MRI knees were performed using the standard Picture Archiving and Communication System (General Electric, New York). Sagittal T2-weighted images of the affected knee was first used to identify the axis of the pes tendons, usually found near the subcutaneous level of the medial tibial plateau (Fig. 2A). The axis of the pes tendons varies from patient to patient (Fig. 2B). In keeping with the same sagittal sequences, sagittal cuts were scrolled laterally along the medial tibial plateau—typically about 12 mm to 21 mm (four to seven sagittal cuts, each cut is 3 mm) laterally from the sagittal view where the axis of the pes tendons were obtained from, until a clear demarcation can be appreciated between the cartilage and the subchondral bone of the medial tibial plateau. We define this as the Liau subchondral bony ridge, which may be present on one to three consecutive sagittal cuts (each cut is 3 mm) (Fig. 3A). An annotation line—line A, parallel to the axis of the pes tendons was placed originating from the ridge on the posterior border of the subchondral surface of the tibial plateau (just anterior to the downward-curving section to the tibial plateau) and extending proximally by 30 mm (Fig. 3A). From the proximal aspect of this 3 cm line, the closest axial MRI cut is used. From this axial cut, cross-sectional lengths and breadths of the semitendinosus tendon and gracilis tendon were determined from magnified axial cuts (Fig. 3B & C). The magnification on screen should be at least ten times the actual cross-sectional lengths and breadths, i.e., a 4 mm length would be 4 cm on screen. In cases where the zone of transition greys between tendon and subcutaneous tissues, measurements were taken at the midpoint of the zone between the clear edge of the tendon and the clear edge of subcutaneous tissues. We specifically used 1.5T MRI knowing that this is the more common MRI standard, and we hoped that this would make our method more convenient and accessible to any orthopedic team. However, if the team would like to use a 3T machine, it will likely be more accurate and the sensitivity and specificity will be even higher.

For line A, the axis along the pes tendons was chosen as we were harvesting these tendons eventually. We noticed there was a variation to the axes of tendons amongst individuals, with some pes tendons being at a more acute angle of pull compared to others. Hence, it was important to individualize and reference from this axis. Regarding the Liau subchondral bony ridge, it was chosen as a reproducible bony landmark that will approximately be at the level of the intraarticular opening of the tibial tunnel. The length of the pes tendons from the level of its tibial insertion to the level of the Liau ridge would therefore approximate the length of the ACL graft in the tibial tunnel, save for a few millimeters due to the oblique tibial insertion of the pes tendons. Reported average intraarticular ACL graft end-to-end lengths were 30.75 mm [14]. Hence, we chose to use a 3 cm proximal distance from the Liau ridge, which would correspond to the level of the pes tendons that, when folded on itself at that level, would be inserted into the femoral tunnel. The dimensions of the semitendinosus and gracilis were then measured from this level. Our method takes into account the noncircular/ovality profile of tendons and utilizes simple measurements of cross-sectional lengths and breadths of the hamstring tendons on the MRI. As this is a retrospective review, the surgeons were all blinded to the above-mentioned measurements.

### Calculation of prediction of graft size

**Fig. 4:**
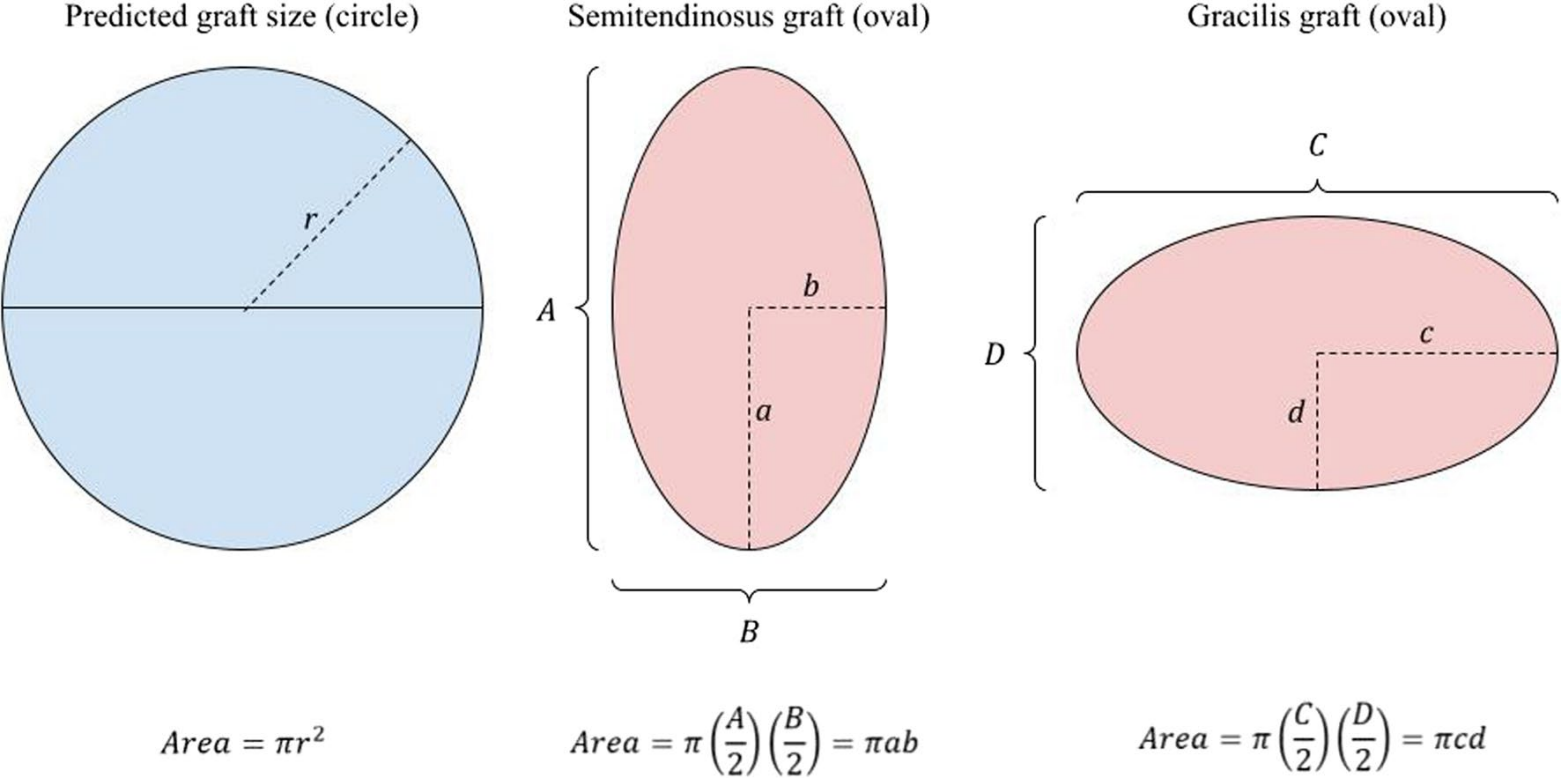
Proposition on shape of predicted graft tendon, based on harvested semitendinosus and gracilis grafts

**Fig. 5:**
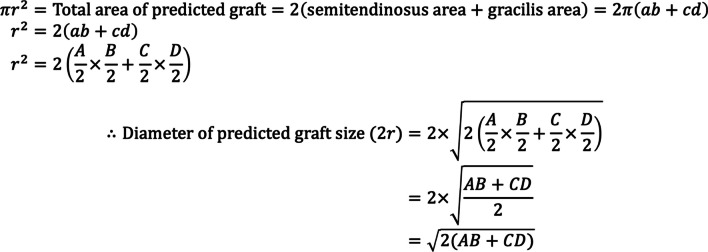
Mathematical proof of proposition

Calculation of diameter of predicted graft size was based on cross sectional measurements of semitendinosus and gracilis tendons (Fig. 4). Cross-sectional lengths and breadths of both semitendinosus and gracilis grafts were recorded with diameter of predicted graft size calculated based on the calculations as shown (Fig. 5). The radius (*r*) of the predicted graft was determined using a circular graft and doubled to obtain the diameter of predicted graft size of a quadrupled hamstring graft. For ease of use, an automated calculator in Microsoft Excel (Additional file 1) was devised to generate the diameter of the predicted graft size from measurements of both hamstring tendons.

An accurate predicted graft size is defined as ± 0.5 mm of the predicted graft size, as the intraoperative graft size measurements are in 0.5 mm increments. For our power analysis, we have defined a power of 80% and alpha of 0.05. Based on existing literature [13] the predicted hamstring cross-sectional area (CSA) as determined by preoperative MRI is 47.2 (± 8.3) mm [8], while the actual CSA determined intraoperatively is 50.01mm^2^. This gives us a sample size of 68.

### Statistical analysis

All statistical analyses were conducted in R (R 4.1.0). The means and 95% confidence intervals (CI) were computed for all continuous variables. The Pearson’s correlation coefficient was calculated accordingly. Specificity and sensitivity was calculated with a confusion matrix. Logistic regression and receiver-operating characteristic curves were computed accordingly. No missing data were encountered throughout the study. Ethics approval has been obtained from the National Healthcare Group (NHG) Domain-Specific Review Board (DSRB no. 2021/00555).

## Results

### Predicting ACL hamstring graft size

A total of 112 patients were recruited for this study. Table 1 presents the cross-sectional MRI measurements of the semitendinosus (SemiT) and gracilis tendons, the predicted quadrupled hamstring graft diameter as calculated from the MRI measurements (Figs. 4 and 5), and the actual quadrupled hamstring graft diameter when measured intraoperatively.

**Table 1:** MRI measurements of tendons, and the predicted and actual graft diameters (*n* = 112). The values are indicated as mean (95% CI)

	Cross sectional measurements done on MRI (mm)				Diameter (mm)	
	SemiT, larger diameter	SemiT, smaller diameter	Gracilis, larger diameter	Gracilis, smaller diameter	Predicted graft diameter	Actual graft diameter
Senior orthopedic resident	5.1 (5–5.3)	3.8 (3.7–3.9)	4.5 (4.3–4.6)	2.7 (2.6–2.8)	7.9 (7.8–8)	7.7 (7.6–7.9)
Junior medical officer	5 (4.9–5.2)	3.6 (3.5–3.7)	4.3 (4.1–4.4)	2.6 (2.5–2.7)	7.6 (7.5–7.8)	7.7 (7.6–7.9)
Average	5.1 (5–5.2)	3.7 (3.6–3.8)	4.4 (4.2–4.5)	2.6 (2.6–2.7)	7.8 (7.6–7.9)	7.7 (7.6–7.9)

### Correlation between the predicted and actual graft size

**Fig. 6:**
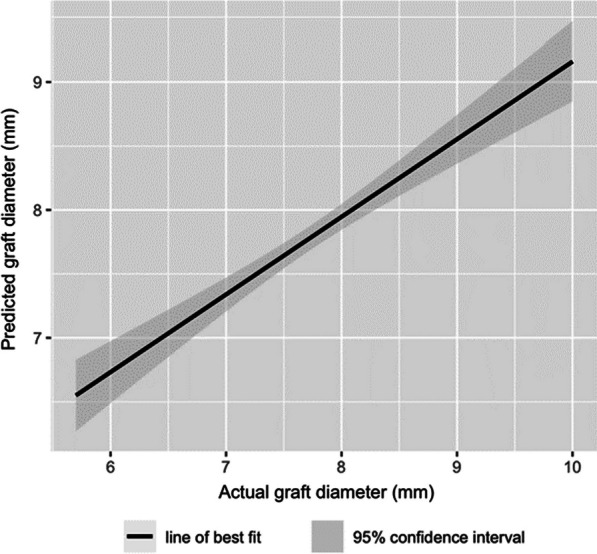
Predicted graft diameter versus actual graft diameter showing a moderate positive correlation

The Pearson’s correlation coefficient for the predicted graft diameter to the actual graft diameter was 0.661 (*p* < 0.001), which shows a moderate positive correlation (Fig. 6).

### *Performance of our method of prediction when defining an adequate actual graft size as *≥ *8 mm*

Literature suggests a higher risk of graft failure or rerupture if the graft diameter is less than 8 mm [3, 13]. Hence, we defined an adequate actual graft diameter as ≥ 8 mm. Using this predicted graft diameter as a cutoff to classify patients (Table 2), 39 out of the 58 patients with adequate actual graft diameters (≥ 8 mm) have been correctly classified, giving a 67.2% sensitivity. At the same time, 50 out of the 54 patients with inadequate actual graft diameters (< 8 mm) have been correctly classified, giving a 92.6% specificity. Our method has a positive predictive value (PPV) of 90.7% and a negative predictive value (NPV) of 72.5%.

**Table 2:** Confusion matrix showing the number of patients correctly classified as having an adequate (≥ 8 mm) or inadequate (< 8 mm) actual graft diameter

	Actual graft diameter (mm)	
	< 8	≥ 8
Predicted graft diameter (mm)		
< 8	50 (92.6%)	19 (32.8%)
≥ 8	4 (7.4%)	39 (67.2%)

**Fig. 7:**
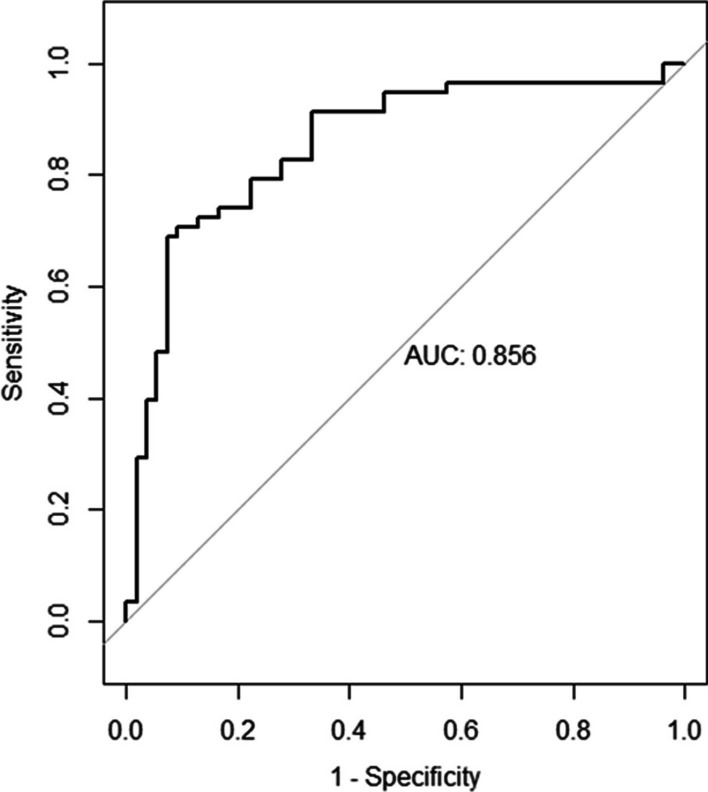
Area under receiver-operating characteristic (ROC) curves plotted with the respective logistic regression models showing good discrimination. Adequate actual graft diameter defined as ≥ 8 mm

A logistic regression model was performed to predict whether the actual graft diameter is adequate (≥ 8 mm) given the predicted graft diameter. The model is significant (*p* < 0.001). The odds of the actual graft diameter being adequate increases by 12.8 for each additional mm of the predicted graft diameter [95% CI (5.2–38.2)]. An area under receiver-operating characteristic (ROC) curve was plotted for the regression model, which shows good discrimination (AUC = 0.856) (Fig. 7).

### Interobserver reliability of prediction method

Lastly, we evaluated the interobserver reliability of the semitendinosus and the gracilis tendon measurements between our two independent blinded evaluators, namely the senior orthopedic resident and the junior medical officer (Table 1). More specifically, we evaluated the extent of agreement in terms of the number of patients who were correctly classified as having either an adequate (≥ 8 mm) or inadequate (< 8 mm) actual graft diameter. Our results show that a high percentage agreement of 83.9% was achieved with a Cohen’s kappa of 0.654, which corresponds to a “substantial” agreement between the two evaluators.

## Discussion

Anthropometric parameters have been shown to not be a reliable tool to predict the intraoperative graft size [15]. Instead, preoperative MRI size measurements of the hamstring tendons have been proven to assist and better predict the intraoperative graft size, with varying sensitivities/specificities ranging from 79%/78% to 81.8%/100% [8–10, 12, 13, 16–18]. This provides the surgical team the ability to adequately prepare beforehand in the event that they may require more adjunctive measures to reinforce the hamstring graft, or to use a different graft altogether. For instance, if the graft size is predicted to be too small, the surgeon can consider to triple the hamstring graft, or even use a different autograft altogether such as a bone–patellar tendon–bone graft or a quadriceps tendon graft. On the other hand, if the graft size is predicted to be large, the surgeon will have even greater confidence that a doubled hamstring graft is sufficient for the patient, and there will be a lower chance of graft failure. This will produce more accurate costing quotations.

### Predicting ACL hamstring graft size

To the authors’ knowledge, this is the first method that allows the predicted graft sizes to be calculated as a continuous numerical manner, as opposed to a dichotomous result. Other methods only provide a dichotomized outcome of whether a graft is sufficient, using a cutoff surface area based on MRI measurements [3, 9, 13, 15]. In comparison, because of our novel mathematical proposition as previously demonstrated, our method is the first method that allows for the user to determine the likely exact value of the axial graft diameter, which the other methods mentioned are not able to provide [8–10, 12, 13, 16]. Furthermore, by using both the cross-sectional length and breadth of the semitendinosus and gracilis tendons, our method takes into account the oval profile of these tendons on the MRI slices, and it does not assume the tendons to only have a circular profile. Our method potentially provides much more information to the surgical team about the magnitude of the difference compared to the ideal hamstring size, which would allow them to make a better decision as to whether they would choose a certain graft method over another. It may also provide additional preoperative planning information in terms of the femoral and tibial tunnel sizes needed to be drilled. Additionally, while the equation is simple enough to calculate on a personal handheld device, we have also created an Excel calculator that would automatically calculate the predicted graft size based on the four parameters (semitendinosus cross-sectional length and width and gracilis cross-sectional length and width).

### Correlation between the predicted and actual graft size

To the authors’ knowledge, our paper represents one of the largest populations where a preoperative MRI is used to predict the actual ACL graft size. Although previously published studies report a positive correlation between the predicted CSA on MRI with the actual intraoperative graft diameters, most of these correlations were low to moderate, with a Pearson’s correlation coefficient of 0.419 [8], 0.495 [16], 0.536 [12], 0.56 [10], 0.641 [9], and 0.697 [19]. In comparison, the Pearson’s correlation coefficient between predicted and actual graft diameter for our method was 0.661 (*p* < 0.001), which is a moderate positive correlation that is superior to previously published studies except Hamada et al. [19]. This could be attributed to the level where we measure the dimensions of the semitendinosus and the gracilis tendons. Other studies which reported a poorer correlation measured the CSA of the tendons at the musculotendinous junction [8], the widest point of the medial femoral condyle [10, 12], or at the physis/physeal scar of the femur [9, 16]. On the other hand, our study measured the tendons 3 cm proximal from the Liau ridge, which is approximately where the ACL graft would be inserted into the femoral tunnel. No other study has taken this anatomical consideration into account, and this might have contributed to the superior correlation. Even though Hamada obtained a marginally better correlation, Hamada used a special image analysis software to count the number of pixels on the axial MRI image to predict the CSA, while our method does not require any specialized software.

### *Performance of our method of prediction when defining an adequate actual graft size as* ≥ *8 mm*

Previously published methods report varying sensitivities/specificities ranging from 79%/78% to 81.8%/100% [8–10, 12, 13, 16–18]. Our method yields a high specificity of 92.6% if we define an adequate actual graft size as ≥ 8 mm, which is noninferior to the previously published methods in terms of specificity and sensitivity. However, previously published methods require specialized tools; Leiter et al. [16] used an exclusive image analysis software to measure the GT and ST CSA, while Wernecke et al. [10], Erquica et al. [12], Beyzadeoglu et al. [8], and Bickel et al. [9] utilized a freehand region-of-interest lasso tool for the measurement of tendon CSA, both of which are not widely available in many PACS systems, nor within our local hospitals. Our method in contrast does not require any specialized software. By simply measuring the larger and smaller diameter of the GT and ST, at this point we provide a significantly more convenient option for hamstring graft size prediction. Additionally, most other papers conducted measurements with experienced radiologists or senior orthopedic surgeons which may not be clinically practical especially in a busy practice. With a high percentage agreement of 83.9% and a Cohen’s kappa of 0.654 corresponding to a “substantial” agreement between our two evaluators, namely a junior medical officer and a senior orthopedic resident, our method has been shown to work accurately even with junior surgical trainees, providing the medical team greater ease of hamstring graft size prediction. Lastly, we recognize the recent trend of “all-inside” technique of ACL reconstruction that utilizes a triple or quadruple semitendinosus graft without the gracilis tendon [20]. However this is not standard practice in our institution, and none of the patients in this study was operated on using this technique.

### Limitations

The limitations of our study include: (1) not considering the variation in degree of knee flexion when the MRI was taken; however, this limitation was not described in other papers and does not seem to have an effect on the accuracy of our results. In addition, we have ensured all MRI scans were performed using one single 1.5T Siemens MAGNETOM Aera scanner (Erlangen, Germany); (2) there may be the use of 3T MRIs in clinical practice, as this would likely increase the accuracy of our measurements, thus resulting in possibly a higher accuracy of predicted graft sizes; (3) the sensitivity of our study is 67.2%, meaning 67.2% of all patients with an adequate graft diameter of ≥ 8 mm were correctly predicted with our method; however sensitivity is less important, since regardless of their predicted graft diameter, this group of patients already have an actual graft diameter that is accurate, (4) Our study is better at predicting grafts that might be too small intraoperatively, compared with predicting grafts that might be too big; however, clinically, it is worse to have a graft that is insufficiently sized compared to a graft that is too large; (5) 4 out of 54 patients with grafts that are too small intraoperatively were unfortunately not picked up with our method; however, this could be attributed to either the grafts being truncated during harvesting resulting in a smaller actual graft size or inconsistent sizing methods depending on how hard the surgeon or the assistant pulls the graft through the sizing cylinders or tubes.

## Conclusions

We present a modified, practical method to predict the ACL hamstring graft size using preoperative MRI measurements that does not require any specialized software or methods and can be reliably done even by junior members of the surgical team. Our results reflect a high specificity of 92.6%, which is a reliable method to assist the surgical team in discussing the appropriate graft options for the patient and to facilitate in preoperative planning.

### Supplementary Information

**Additional file 1.** An automated calculator in Microsoft Excel that generates the diameter of the predicted graft size from the measurements of the semitendinosus and gracilis tendons.

## Data Availability

The datasets used and/or analyzed during the current study are available from the corresponding author on reasonable request.
